# Feasibility of intraoperative force quantification during robotic paraesophageal hernia repair: a prospective case series using integrated force-sensing technology

**DOI:** 10.3389/fsurg.2026.1815403

**Published:** 2026-05-20

**Authors:** Emily P. Rabinovich, Lauren Carroll, Donovan Hui, Cullen Carter, Peter T. Hallowell, Claire Foley, Allan Tsung, Thomas H. Shin

**Affiliations:** Department of Surgery, University of Virginia Health System, Charlottesville, VA, United States

**Keywords:** force feedback, foregut surgery, paraesophageal hernia repair, robotic surgery, surgical approach standardization, surgical biomechanics, training

## Abstract

**Background:**

Paraesophageal hernia repair (PEHR) is associated with high complication rates and 5-year recurrence rates of 30%–66%, with excessive tissue tension and trauma hypothesized as key contributors. However, intraoperative assessment remains qualitative, relying on surgeon perception rather than objective measurement. This study evaluates the feasibility of applying integrated force-sensing robotic technology to perform prospective, phase-segmented analysis of intraoperative tissue forces during PEHR and characterize force variability across defined operative phases.

**Methods:**

A prospective evaluation of integrated force-sensing technology was conducted across nine consecutive robotic PEHRs performed at a single academic center on the da Vinci 5 robotic platform. Force data were collected from two Cadiere forceps and one Mega SutureCut needle driver. Operative workflow was segmented *a priori* into four biomechanically relevant phases: mediastinal dissection, crural tension assessment, cruroplasty, and fundoplication. Primary outcomes included mean force per phase (Newtons) and variability across cases. To assess the discriminative capacity of the force measurement framework, exploratory between-case comparisons were performed using two-tailed t-tests with Cohen’s d effect size estimation against a designated low-complexity reference case. All cases were performed by fellowship-trained robotic foregut surgeons using a standardized technique with uniform haptic sensitivity settings.

**Results:**

Force data were successfully captured across all nine cases for all operative phases performed in each case, with 832–2,297 measurements per phase. Distinct phase-specific force profiles were observed: mediastinal dissection (2.00–3.04N), crural tension assessment (1.50–3.35N), cruroplasty suturing (1.68–3.58N), and fundoplication (1.96–2.97N), which demonstrated the greatest consistency. Exploratory comparisons demonstrated that the force measurement framework detected statistically distinguishable force profiles between cases of varying complexity (*p* < 0.001, Cohen’s d: 0.21–0.30), including among both complicated and uncomplicated cases. Five patients experienced perioperative complications, including pleural injuries, dysphagia, and one recurrence.

**Conclusion:**

Quantification of phase-specific intraoperative forces during robotic PEHR is feasible using integrated force-sensing technology. Observed variability across operative phases and the ability to detect distinguishable force profiles across cases of varying complexity support further investigation to define clinically meaningful force thresholds and potential applications in surgical standardization and training.

## 1 Introduction

Paraesophageal hernia repair (PEHR) remains technically challenging, with recurrence rates reported between 30%–66% (1, 2). Excessive tissue tension and trauma have been proposed as key drivers of postoperative complications and recurrence, influencing outcomes across the spectrum of repair techniques from crural closure strategy to mesh reinforcement decisions (3–6). However, intraoperative assessment of these factors remains largely qualitative, relying on surgeon gestalt and experience rather than objective measurement (3, 4, 7, 8). This gap between the recognized importance of tension and the inability to measure it has limited the development of evidence-based criteria for repair technique selection and intraoperative decision-making.

Advances in minimally invasive robotic surgery have improved visualization, instrument articulation, and patient outcomes, but robotic platforms have historically lacked tactile feedback, requiring surgeons to rely on visual cues rather than haptic perception during tissue manipulation. The recent introduction of integrated force-sensing instrumentation on robotic surgical platforms now enables both real-time haptic perception and, critically, the quantification and recording of instrument-tip forces during surgery (9). Pre-clinical studies using this technology have demonstrated that force feedback reduces applied tissue forces and improves suturing performance (5, 6), and recent clinical studies have reported aggregate force data across thoracic and colorectal procedures (10, 11). However, no study has applied prospective, phase-segmented force analysis to characterize the distinct biomechanical demands of individual operative phases during PEHR, a procedure in which tissue tension is hypothesized to directly influence repair durability and complication risk.

The present study reports a prospective case series evaluating the feasibility of capturing and characterizing phase-specific intraoperative force metrics during robotic PEHR using the da Vinci 5 force-sensing platform. Operative workflow was deconstructed *a priori* into four biomechanically relevant phases to enable granular characterization of force dynamics across distinct modes of tissue interaction: mediastinal dissection, crural tension assessment, cruroplasty, and fundoplication. The primary aim was to establish that intraoperative force measurement during PEHR is feasible and generates reproducible, phase-specific force data. Secondary exploratory aims included characterizing force variability across cases and operative phases and assessing whether the force measurement framework could discriminate between cases of varying operative complexity. By defining baseline force metrics for each operative phase, this work represents an initial step toward the objective deconstruction and standardization of surgical technique in PEHR where the two hypothesized drivers of repair failure, tissue tension and trauma, have until now been unmeasurable in the robotic approach.

## 2 Methods

A prospective case series of nine consecutive robotic PEHR procedures performed using integrated force-sensing instrumentation on the da Vinci 5 system (Intuitive Surgical, Sunnyvale CA) was conducted at a single tertiary academic center between February 2025 and October 2025. The study was designed as an exploratory investigation to characterize intraoperative surgical biomechanics via quantitative force measurements and to begin identifying reproducible force signatures across predefined operative phases. The University of Virginia Institutional Review Board reviewed and approved this study (IRB HSR #302016) and granted a waiver of informed consent given the minimal-risk nature of the data collection, which involved recording de-identified instrument force data generated during standard surgical care. No experimental interventions were performed; all surgical decisions were made independently of the force data.

**Figure 1:**
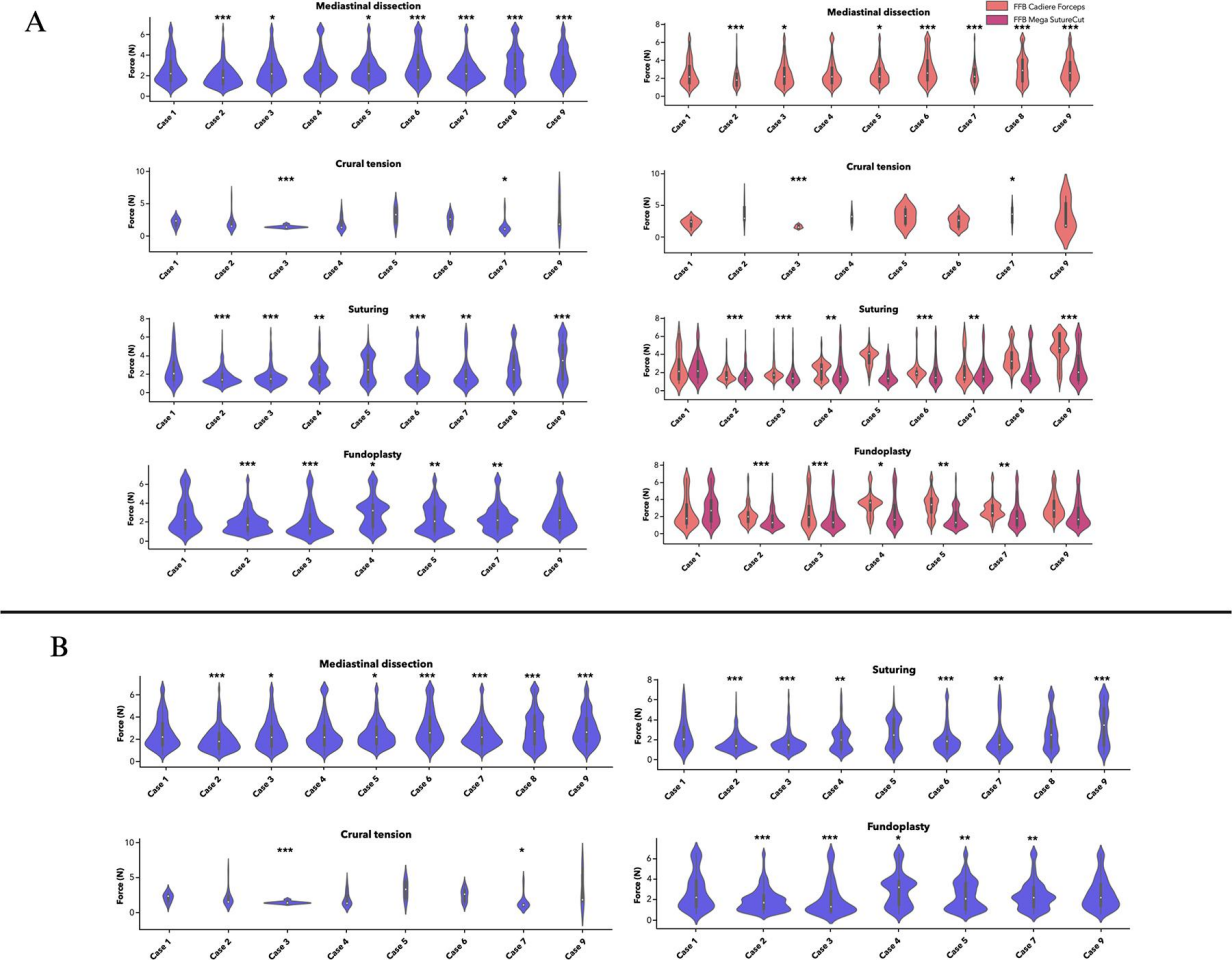
Intraoperative force distributions during robotic paraesophageal hernia repair. **(A)** Force distributions by operative segment separated by instrument type (Cadiere forceps vs. Mega SutureCut needle driver) across nine consecutive robotic paraesophageal hernia repairs. **(B)** Force distributions by operative segment across the same cohort, comparing cases relative to the reference case (Case 1) to illustrate measurement discrimination across cases of varying complexity. Force measurements per segment ranged between 832 and 2297 measurements. Case 1 served as the reference case. Cases 6–9 demonstrated significant postoperative complications (see Table 2). *: *p* < 0.05, **: *p* < 0.01, ***: *p* < 0.001. (Created by E.P.R. in https://BioRender.com). Violin plots display the kernel density estimation of force distributions for each case within each operative phase, with wider sections indicating higher measurement density at that force level. The white dot indicates the median, the thick bar represents the interquartile range, and the thin line extends to 1.5× the interquartile range.

Force data were captured using two Cadiere forceps and one Mega SutureCut needle driver with integrated force sensors that measure directional forces at the instrument tip. The sensors are calibrated for force measurements within the range of 0.6–6.5 N, with verified accuracy within this operating range. When applied forces exceed 6.5 N, stress shielding within the instrument may lead to underreporting of true applied force; readings at or above 6.5 N should therefore be interpreted as a lower bound rather than a precise measurement. Force metrics reported in this study including mean force, standard deviation, median, and interquartile range were derived from computed force metrics sampled at 50 Hz, which provide accurate measurements within the 0.6–6.5 N calibrated range. Additionally, 1 Hz force timeseries data were used to characterize peak force profiles and distributional features visualized in Figure 1. As a result, distributional metrics below the sensor ceiling, including median force and interquartile range, provide more informative characterization of force profiles than peak force alone. Force data timestamps were aligned with operative video recordings to enable accurate assignment of force measurements to predefined operative phases. Phase segmentation was performed by fellowship-trained surgeon investigators through structured video review of each case. Operative workflow was segmented *a priori* into four biomechanically relevant phases each defined by procedural and anatomic landmarks to capture distinct types of tissue interaction: mediastinal dissection, crural tension assessment (defined as the phase capturing forces during manual reapproximation of the crura across the hiatal defect prior to suture repair), cruroplasty, and fundoplication (Table 1). Each operative phase encompasses multiple distinct surgical actions (e.g., grasping, retracting, dissecting, suturing) that generate heterogeneous force types including tension, compression, and shear. Phase-level force metrics therefore represent composite summaries of instrument-tissue interaction across these actions. This level of segmentation was selected as the appropriate granularity for an initial feasibility study; finer decomposition into individual surgical actions (e.g., discrete grasping events, individual suture throws, traction maneuvers) requires validated action-annotation frameworks and is planned as a subsequent analytic step within a hierarchical decomposition approach. Segmentation was independently cross validated against instrument force data logs by technical personnel at Intuitive Surgical, who had no role in study design, data interpretation, or manuscript preparation.

**Table 1:** *A priori* operative phase segmentation with procedural and anatomic landmarks for force data assignment during robotic paraesophageal hernia repair.

Case Segment	Start Landmark	End Landmark
Mediastinal Dissection	Right crus identification	Mediastinal dissection complete
Crural Tension	Initial grasping of contralateral crura with each Cadiere forceps	Completion of crural reapproximation
Cruroplasty	First Cruroplasty suture initiated	Last Cruroplasty suture cut
Fundoplication	Start of shoeshine maneuver	Last fundoplication suture cut

The primary outcome was the feasibility of capturing phase-specific force data during robotic PEHR, assessed by successful force recording across all operative phases. Primary force metrics included mean force per instrument within each operative phase with standard deviation. To provide fuller distributional characterization, median force with interquartile range (IQR) are also reported per instrument per phase (Supplementary Table S1). Given the 6.5 N sensor ceiling, peak force analysis is limited to phases where maximum recorded forces fall below the truncation threshold, most notably crural tension assessment where peak forces reflect native tissue characteristics and vary across cases. Force distributions were summarized using descriptive statistics and visualized using violin plots to characterize distributional features.

To assess whether phase-specific force measurement could detect between-case differences in operative force profiles, Case 1 was designated as a low-complexity reference case based on its uncomplicated clinical course, absence of reoperative anatomy, and small hernia size. Two-tailed t-tests with Cohen’s d effect size estimation were used for pairwise comparisons of mean phase-specific forces between individual cases and the reference case. These comparisons are intended to evaluate the discriminative capacity of the force measurement framework across cases of varying complexity rather than to establish normative force thresholds or test force-outcome hypotheses and should be interpreted as descriptive rather than confirmatory. Given the large number of non-independent, temporally autocorrelated force measurements per operative phase, statistical significance is expected even for small absolute differences and reported *p*-values are likely inflated by pseudoreplication. Effect sizes (Cohen’s d) are therefore reported alongside *p*-values to provide a more meaningful characterization of the magnitude of observed differences. No power calculation was performed as this was an exploratory feasibility study designed to establish proof-of-concept and the sample of nine consecutive cases reflects the available cohort during the study period.

De-identified patient demographics, hernia characteristics (type, size, reoperative status), surgical techniques (cruroplasty method, mesh use), and perioperative complications were collected. Complications were classified as intraoperative (e.g., pleural injury, vascular injury) or postoperative (e.g., dysphagia, hernia recurrence) and are reported descriptively. All procedures were performed by four fellowship-trained robotic foregut surgeons using a standardized operative approach and uniform system settings, including medium haptic sensitivity, to minimize intersurgeon technical variability. This study was reported in accordance with STROBE guidelines.

## 3 Results

Table 2 summarizes patient demographics, hernia characteristics, and operative variables across nine consecutive robotic PEHR cases. The cohort included Types I–III paraesophageal hernias (greatest diameter 2–5.5 cm), two reoperative repairs, and varied cruroplasty techniques including one case with biologic mesh reinforcement. Five cases had perioperative complications: three intraoperative pleural injuries (Cases 6, 7, 8), one with concurrent vascular injury (Case 6); postoperative dysphagia in three cases (Cases 6, 8, 9); one hernia recurrence at postoperative day 70 (Case 6); and one pulmonary embolism on postoperative day 2 (Case 4), which was considered unrelated to intraoperative tissue handling. Complication severity ranged from self-limited pleural tears requiring no intervention to major vascular injury and hernia recurrence.

**Table 2:** Patient and hernia characteristics across nine consecutive PEHR cases.

Case	Patient Characteristics	PEHR	Hiatal Repair Technique	Complication(s)	Mediastinal dissection force (*N* ± SD)	*p*-value*
Case 1	68-year-old male, BMI 28, previous pulmonary embolism, obstructive sleep apnea, esophagitis	Type 1; 2 × 1.5cm	Running posterior 2–0 v-loc PBT	None	2.59 ± 1.43	N/A
Case 2	53-year-old female, BMI 27	Type 1; 3 × 2cm	Running anterior and posterior 2–0 v-loc PBT	None	2.00 ± 1.09	<0.001
Case 3	43-year-old male, BMI 30	Type 1; 3 × 1.5cm	Running posterior 2–0 v-loc PBT	None	2.47 ± 1.35	0.02
Case 4	48-year-old female, BMI 34, asthma, gastric volvulus reduced preoperatively, previous robotic salpingo-oophorectomy	Type 1; 4 × 2.5cm	Running posterior 2–0 v-loc PBT	POD2 segmental pulmonary embolus, numerous ED visits for chest pain up to POD82	2.58 ± 1.47	0.78
Case 5	62-year-old female, BMI 30, smoker, esophagitis, previous laparoscopic cholecystectomy	Type 1; 5 × 2.5cm	Running posterior 2–0 v-loc PBT	None	2.47 ± 1.18	0.02
Case 6	75-year-old male, BMI 29, ascending aortic aneurysm	Type 2; 5.5 × 2cm	Interrupted 2–0 Ethibond with biologic mesh	Right pleural tear with hypotension and arterial bleed; POD7 dysphagia, POD28 mediastinal collection, POD70 recurrence	3.04 ± 1.57	<0.001
Case 7	56-year-old female, BMI 40, breast cancer, anemia from Cameron’s ulcers	Type 3; 5 × 2.5cm	Running posterior 2–0 v-loc PBT	Right apical pleural tear	2.43 ± 1.19	<0.001
Case 8	48-year-old female, BMI 23, obstructive sleep apnea, congestive heart failure, reflux, hypertension, numerous prior laparoscopic abdominal surgeries	Type 1; 3 × 2 cm; 3rd recurrence	Vertical mattress 0-Ethibond with pledgets	Right pleural tear with large venous injury; POD14 dysphagia (ongoing) with paroxysmal atrial tachycardia	2.95 ± 1.64	<0.001
Case 9	45-year-old male, BMI 30, slipped Nissen with recurrence, numerous prior open abdominal surgeries	Type 3; 3 × 1 cm; 2nd recurrence	Running posterior 2–0 v-loc PBT	POD9 dysphagia (ongoing)	2.90 ± 1.42	<0.001

BMI, body mass index; PBT, polybutester; POD, postoperative day; N, newton; SD, standard deviation.

* *p*-values from exploratory comparisons against Case 1 (low-complexity reference) to assess measurement discrimination.

Force data were successfully captured across all nine cases, with 832–2,297 force measurements recorded per phase segment (Figures 1A,B). Not all four phases were performed in every case; fundoplication was not performed in two cases based on clinical decision-making, and these cases were excluded from fundoplication force analysis. Instrument force profiles varied by operative phase, with distinct distributional characteristics observed across the four modes of tissue interaction. Across all cases and phases, force distributions were non-uniform, characterized by intermittent high-force events rather than sustained continuous loading.

During mediastinal dissection, mean forces ranged from 2.00 to 3.04 N (SD: 1.09–1.64 N). In exploratory comparisons designed to assess measurement sensitivity, the force framework detected statistically significant differences between individual cases and the reference across a range of case complexity. Cases 6, 8, and 9 (all with perioperative complications) demonstrated higher forces relative to the reference (*p* < 0.001, Cohen’s d: 0.21–0.30), while Cases 2 and 5 (both uncomplicated) also reached significance (*p* < 0.001 and *p* = 0.02, respectively), confirming that the measurement system discriminates force profiles independently of complication status. Within-phase standard deviations (1.09–1.64 N) reflect the magnitude of force fluctuation during dissection, consistent with episodic high-force events interspersed with lower-force tissue manipulation; full distributional metrics including median and interquartile range are reported in Supplementary Table S1. Cruroplasty suturing forces demonstrated the greatest between-case variability (1.68–3.58 N, SD: 0.90–2.07 N). Forces were significantly lower in Cases 2 and 3 (both uncomplicated, *p* < 0.001) and significantly higher in Case 9 (complicated, *p* < 0.001) relative to the reference. Within-phase standard deviations during cruroplasty were the highest among all phases (0.90–2.07 N), reflecting substantial force fluctuation consistent with the varied loading demands of suture placement and tensioning (Supplementary Table S1). Fundoplication demonstrated the most consistent force profiles across the seven cases in which fundoplication was performed (1.96–2.97 N, SD: 1.18–1.79 N), with minimal statistically significant differences from the reference case. Within-phase standard deviations during fundoplication (1.18–1.79 N) were narrow relative to other phases, consistent with the uniform force fluctuation profile expected from a standardized reconstructive step (Supplementary Table S1).

Crural tension assessment, which captures forces during manual reapproximation of the crura prior to suture repair and reflects native hiatal defect characteristics rather than surgical technique, demonstrated the lowest overall mean forces (1.50–3.35 N, SD: 0.18–2.23 N). Force variability across cases during this phase was wide, consistent with the heterogeneity of hernia size and defect morphology in the cohort. Among the four operative phases, crural tension assessment demonstrated the greatest between-case variability in peak force, with maximum recorded forces ranging from 2.0 to 6.5 N. The majority of cases remained below the 6.5 N sensor ceiling during this phase, in contrast to mediastinal dissection, cruroplasty, and fundoplication where peak forces reached the ceiling in nearly all cases. Crural tension assessment is therefore the phase in which peak force most directly reflects case-specific anatomy rather than instrumentation constraints, and where peak force analysis is most informative for characterizing between-case differences in native tissue tension.

## 4 Discussion

This study demonstrates the feasibility of quantifying phase-specific intraoperative force metrics during robotic PEHR using integrated force-sensing instrumentation. Force data were successfully captured across all nine cases and completed predefined operative phases, with sufficient measurement density (832–2,297 measurements per phase) to characterize distributional features of instrument-tissue interaction. Distinct force profiles were observed across operative phases, with mediastinal dissection and cruroplasty demonstrating greater between-case variability than fundoplication, which showed the most consistent force profiles. Across all phases, force distributions were non-uniform, characterized by intermittent high-force events rather than sustained loading. This suggests that surgical tissue interaction during PEHR is episodic rather than continuous, a finding with potential implications for how force thresholds and tissue trauma are defined in future studies.

The *a priori* segmentation of operative workflow into biomechanically relevant phases revealed that different phases capture fundamentally different types of information. Mediastinal dissection and cruroplasty forces reflect active surgical technique and tissue handling, where force variability between cases may represent differences in dissection approach, tissue quality, or case complexity. Fundoplication forces were the most uniform, likely reflecting the relatively standardized nature of this reconstructive step. Among the four phases, crural tension assessment is unique in that it reflects native tissue characteristics rather than surgical technique; specifically, the force required to reapproximate the crura across the hiatal defect. The hypothesis that higher baseline crural tension correlates with increased probability of hernia recurrence is mechanistically plausible, as greater tension on the cruroplasty repair is a recognized risk factor for repair failure (3–5). While the present cohort is too small to test this hypothesis, the ability to objectively quantify pre-repair crural tension represents a potential application of force-sensing technology for intraoperative decision-making (i.e., whether primary crural closure alone is sufficient or whether mesh reinforcement or a relaxing incision is warranted based on a measured tension threshold rather than subjective assessment).

In exploratory comparisons, the force measurement framework detected statistically distinguishable force profiles across cases of varying complexity, with both complicated and uncomplicated cases demonstrating significant differences relative to the reference. An important limitation in interpreting these associations is that elevated intraoperative forces may serve as a marker of case complexity rather than an independent causal contributor to complications. Patients with larger hernias, reoperative anatomy, or inflamed tissue require greater dissection force, and these same factors independently increase complication risk. The present study cannot distinguish between force as a mediator of tissue injury vs. force as a surrogate marker of operative difficulty, and disentangling this relationship will require larger, controlled studies with standardized case-mix adjustment.

A related consideration is whether the adoption of robotic surgical platforms without tactile feedback introduces safety trade-offs relative to open and laparoscopic approaches where surgeons retain direct haptic perception. The high complication rate observed in this series (five of nine cases) warrants transparent discussion, though the cohort included two reoperative repairs and complex hernia morphology, and complication severity ranged from self-limited pleural tears to major vascular injury and recurrence. Published series of paraesophageal hernia repairs report complication rates of 5%–28% (1, 12–16); the rate observed in the present series, while high, is consistent with the complexity and reoperative nature of cases in this study. Rather than suggesting that force-sensing technology contributed to complications, the data illustrate the potential value of objective force measurement as a safety-enhancing tool. By converting previously unquantifiable tissue interaction into measurable parameters, force sensing may enable identification of high-risk intraoperative patterns and support the development of force thresholds that guide safer tissue handling across all robotic procedures.

The density and temporal resolution of force data captured in this study create opportunities for advanced analytic approaches, including machine learning algorithms that may identify force patterns predictive of outcomes (17). Future studies should examine whether distributional characteristics such as force variability, peak frequency, or temporal patterns provide additional prognostic information beyond mean force values. Larger, multicenter prospective studies with standardized case-mix data and long-term follow-up are needed to define clinically actionable force thresholds and to validate the association between intraoperative force metrics and outcomes including hernia recurrence. Beyond operative decision-making, objective force measurement has implications for robotic surgical training. Quantitative force sensing introduces the possibility of defining phase-specific biomechanical targets, enabling trainees to calibrate tissue handling against measurable parameters rather than relying solely on subjective feedback. Real-time force monitoring may also reduce technical variability during training by providing immediate feedback when applied force exceeds predefined ranges, potentially mitigating tissue injury associated with the learning curve.

This study must be evaluated in the context of several limitations. The cohort of nine consecutive cases at a single institution limits statistical power and generalizability, precluding determination of definitive force thresholds. The use of a single low-complexity case as a reference point for assessing measurement discrimination, rather than a formal control group, limits the generalizability of between-case comparisons; the reference case was selected to demonstrate that the force framework can detect differences across cases of varying complexity, not to define normative force benchmarks. Future studies should compare complicated and uncomplicated cohorts as groups with appropriate case-mix adjustment. Given the large number of non-independent force measurements per operative phase (832–2,297 per segment), the exploratory statistical comparisons should be interpreted as descriptive rather than confirmatory, as the large number of non-independent, temporally autocorrelated measurements per phase constitutes pseudoreplication that inflates statistical significance. Effect sizes (Cohen’s d) were therefore reported alongside *p*-values to provide more meaningful estimates of between-case differences. Future studies with larger cohorts should employ hierarchical or mixed-effects models that formally account for within-case temporal autocorrelation and clustering at the patient level. Force measurements reflect instrument-tissue interactions at the instrument tip rather than direct tissue stress, and the relationship between applied instrument force and tissue-level biomechanical strain requires further investigation. Furthermore, no validated force thresholds currently exist for PEHR or comparable foregut procedures, and the clinical significance of the reported force ranges, including whether they approximate levels associated with tissue injury, remains to be established. The force values reported in this study should therefore be interpreted as descriptive benchmarks for this procedure and platform rather than as clinically actionable thresholds. Follow-up duration was short and variable, limiting assessment of long-term outcomes including hernia recurrence. Additionally, the integrated force sensors are calibrated for the 0.6–6.5 N range, and stress shielding above 6.5 N may lead to underreporting of true applied force. Analysis of high-resolution (50 Hz) computed force data across all nine cases revealed that force readings exceeding 6.5 N represented a mean of 1.9% of all measurements across cases and operative phases. Truncation was not uniformly distributed across phases. Cruroplasty had the highest proportion of measurements exceeding 6.5 N (3.3%), followed by fundoplication (1.9%) and mediastinal dissection (1.8%), while crural tension assessment had essentially no measurements above 6.5 N (<0.1%), further supporting its suitability for peak force characterization. Truncation was modestly more frequent in complicated cases (2.7%) than uncomplicated cases (1.1%), with the highest proportions observed in Case 8 (8.8% overall), a third-time reoperative repair in which cruroplasty forces exceeded 6.5 N in 19.3% of measurements. When forces did exceed the calibrated range, true peak forces were substantially higher, with maximum recorded forces reaching up to 61.2 N during mediastinal dissection and 24.9 N during cruroplasty. These findings indicate that while truncation affects a small minority of measurements and is unlikely to substantially alter the phase-level distributional characterization reported in this study, the concentration of high-force events in complex cases and during cruroplasty underscores the importance of full-range force measurement for future studies examining force-outcome relationships and tissue injury thresholds. This constrains peak force analysis using the 1 Hz timeseries to phases where maximum recorded forces fall below the 6.5 N threshold, most notably crural tension assessment. The four-phase segmentation employed in this study also aggregates multiple distinct surgical actions within each phase, and phase-level metrics represent composite summaries across heterogeneous force-generating maneuvers including grasping, retraction, and suturing. While this granularity is appropriate for establishing feasibility and generating reproducible phase-specific benchmarks, future studies should pursue finer decomposition into discrete surgical actions using validated action-annotation frameworks to characterize force types associated with specific tissue interactions and to identify which intraoperative force events most directly correlate with tissue injury. Both extended sensor dynamic range and sub-phase temporal resolution will be necessary to define action-specific force thresholds with direct biomechanical correlates. Finally, while four fellowship-trained foregut surgeons performed all cases using a standardized technique and uniform haptic sensitivity settings, intersurgeon variability in tissue handling cannot be fully excluded and should be evaluated in future multi-surgeon studies. Additionally, while phase segmentation was performed by fellowship-trained surgeon investigators using predefined procedural and anatomic landmarks and independently cross-validated by technical personnel at Intuitive Surgical, formal interobserver agreement metrics were not calculated between surgeon reviewers. Future studies should assess segmentation reproducibility using standard measures of interrater reliability to strengthen confidence in the phase-assignment methodology. Despite these limitations, this work establishes feasibility and provides foundational phase-specific force benchmarks to inform the design of larger prospective studies.

## 5 Conclusions

This study demonstrates the feasibility of quantifying phase-specific intraoperative force metrics during robotic PEHR using integrated force-sensing instrumentation, establishing that tissue forces across distinct operative phases can be objectively measured and characterized. Force variability was observed across operative phases and cases, with distinct biomechanical profiles reflecting different modes of tissue interaction and the capacity to discriminate force profiles across cases of varying complexity. These exploratory findings provide foundational benchmarks to support larger prospective studies aimed at defining clinically meaningful force thresholds, investigating the relationship between intraoperative tissue tension and hernia recurrence, and integrating quantitative biomechanical feedback into surgical decision-making and training.

## Data Availability

The raw data supporting the conclusions of this article will be made available by the authors, without undue reservation.
