# Supplementary material for: Feasibility of intraoperative force quantification during robotic paraesophageal hernia repair: a prospective case series using integrated force-sensing technology
Source: Front Surg. 2026 May 20;13:1815403. doi: 10.3389/fsurg.2026.1815403 (PMC13230167; doi:10.3389/fsurg.2026.1815403)
Supplement: Supplementary file 1 [file Datasheet1.pdf]

Supplemental Table 1. Descriptive force metrics per phase and case.

| | Cadiere Forceps | | | Mega SutureCut | | |
|---|---|---|---|---|---|---|
| | Mean ± SD | Median (IQR) | Peak | Mean ± SD | Median (IQR) | Peak |
| | | | **Mediastinal Dissection** | | | |
| Case 1 | 2.59±1.43 | 2.20 (1.50-3.40) | 6.50 | — | — | — |
| Case 2 | 2.01±1.09 | 1.80 (1.20-2.50) | 6.50 | 1.08±0.27 | 0.90 (0.90-1.30) | 1.50 |
| Case 3 | 2.47±1.35 | 2.20 (1.40-3.15) | 6.50 | — | — | — |
| Case 4 | 2.58±1.47 | 2.20 (1.50-3.20) | 6.50 | — | — | — |
| Case 5 | 2.47±1.18 | 2.20 (1.60-3.10) | 6.50 | — | — | — |
| Case 6 | 3.04±1.57 | 2.60 (1.82-4.00) | 6.50 | — | — | — |
| Case 7 | 2.43±1.19 | 2.20 (1.60-3.00) | 6.50 | 0.96±0.38 | 0.80 (0.70-1.07) | 1.70 |
| Case 8 | 3.06±1.58 | 2.90 (1.70-4.10) | 6.50 | 2.14±1.87 | 1.30 (0.80-2.70) | 6.50 |
| Case 9 | 2.90±1.42 | 2.60 (1.80-3.80) | 6.50 | — | — | — |
| | | | **Crural Tension** | | | |
| Case 1 | 2.30±0.63 | 2.50 (1.80-2.65) | 3.30 | — | — | — |
| Case 2 | 3.83±1.46 | 3.05 (2.80-4.80) | 6.50 | 1.64±0.11 | 1.60 (1.60-1.70) | 1.90 |
| Case 3 | 1.70±0.21 | 1.60 (1.50-1.90) | 2.00 | 1.43±0.11 | 1.40 (1.35-1.50) | 1.60 |
| Case 4 | 3.11±0.79 | 3.30 (2.40-3.40) | 4.70 | 1.31±0.10 | 1.30 (1.25-1.30) | 1.50 |
| Case 5 | 3.34±1.20 | 3.40 (2.20-4.40) | 4.90 | — | — | — |
| Case 6 | 2.57±0.91 | 2.70 (1.70-3.30) | 4.20 | — | — | — |
| Case 7 | 3.56±1.10 | 3.70 (2.40-4.60) | 4.90 | 1.18±0.09 | 1.15 (1.10-1.20) | 1.40 |
| Case 8 | — | — | — | — | — | — |
| Case 9 | 3.35±2.23 | 1.90 (1.73-5.38) | 6.50 | — | — | — |
| | | | **Cruroplasty** | | | |
| Case 1 | 2.55±1.63 | 2.10 (1.20-3.50) | 6.50 | 2.58±1.40 | 2.15 (1.50-3.30) | 6.50 |
| Case 2 | 1.73±0.83 | 1.40 (1.20-2.00) | 5.40 | 1.63±0.96 | 1.40 (1.00-1.90) | 6.30 |
| Case 3 | 1.92±0.93 | 1.70 (1.40-1.90) | 6.50 | 1.60±1.12 | 1.30 (0.90-1.80) | 6.50 |
| Case 4 | 2.22±1.01 | 2.40 (1.20-2.90) | 5.50 | 2.09±1.54 | 1.50 (0.97-2.62) | 6.50 |
| Case 5 | 3.77±0.97 | 4.20 (3.10-4.30) | 6.50 | 1.67±0.99 | 1.30 (1.00-2.20) | 4.60 |
| Case 6 | 2.19±1.16 | 1.90 (1.73-2.10) | 6.50 | 2.00±1.51 | 1.40 (0.90-2.50) | 6.50 |
| Case 7 | 2.28±1.67 | 1.40 (1.20-2.70) | 6.50 | 1.96±1.43 | 1.50 (1.00-2.40) | 6.50 |
| Case 8 | 3.67±1.36 | 3.30 (2.50-4.30) | 6.50 | 2.30±1.77 | 1.60 (1.00-3.00) | 6.50 |
| Case 9 | 4.74±1.60 | 4.80 (4.35-6.50) | 6.50 | 2.73±1.97 | 2.00 (1.10-3.90) | 6.50 |
| | | | **Fundoplication** | | | |
| Case 1 | 2.52±1.78 | 1.80 (1.20-3.40) | 6.50 | 2.99±1.77 | 2.70 (1.45-3.90) | 6.50 |
| Case 2 | 2.20±1.15 | 2.00 (1.40-2.50) | 6.50 | 1.67±1.16 | 1.30 (0.80-2.10) | 6.50 |
| Case 3 | 2.52±1.87 | 1.95 (1.00-3.20) | 6.50 | 1.93±1.55 | 1.30 (0.80-2.50) | 6.50 |
| Case 4 | 3.45±1.28 | 3.60 (2.70-3.80) | 6.50 | 2.42±1.85 | 1.60 (1.00-3.30) | 6.50 |
| Case 5 | 3.33±1.38 | 3.40 (2.50-4.10) | 6.50 | 1.81±1.20 | 1.30 (0.90-2.50) | 6.50 |
| Case 6 | — | — | — | — | — | — |
| Case 7 | 2.78±1.08 | 2.40 (2.10-3.30) | 6.50 | 2.20±1.58 | 1.80 (1.00-2.60) | 6.50 |
| Case 8 | — | — | — | — | — | — |
| Case 9 | 3.08±1.38 | 2.70 (2.00-3.80) | 6.50 | 2.25±1.57 | 1.65 (1.10-3.00) | 6.50 |
